# p21-Activated Kinase 4 Promotes Intimal Hyperplasia and Vascular Smooth Muscle Cells Proliferation during Superficial Femoral Artery Restenosis after Angioplasty

**DOI:** 10.1155/2017/5296516

**Published:** 2017-06-19

**Authors:** Liangxi Yuan, Xianli Duan, Jian Dong, Qingsheng Lu, Jian Zhou, Zhiqing Zhao, Junmin Bao, Zaiping Jing

**Affiliations:** Department of Vascular Surgery, Changhai Hospital, Second Military Medical University, Shanghai 200433, China

## Abstract

The aim of this study is to explore the function of p21-activated kinase 4 (PAK4) in intimal hyperplasia (IH) and vascular smooth muscle cells (VSMCs) proliferation. We choose vascular samples from patients undergoing angioplasty in superficial femoral artery (SFA) as the experimental group and vascular samples from donors without clinical SFA restenosis as the control group, respectively. We draw from the results that both levels of mRNA and protein of PAK4 in the experimental group increased dramatically compared with the control group. IH arose from angioplasty of SFA. Moreover, overexpression of PAK4 dramatically contributed to cell proliferation of VSMCs and promoted cell cycle progression from G0/G1 phase (71.12 ± 0.69% versus 58.77 ± 0.77%, *P* < 0.001) into S phase (23.99 ± 0.21% versus 31.35 ± 0.33%, *P* < 0.001). Besides, PAK4 downregulated the level of p21 and enhanced the activity of Akt as well. And we conclude that PAK4 acts as a regulator of cell cycle progression of VSMC by mediating Akt signaling and controlling p21 levels, which further modulate IH and VSMCs' proliferation.

## 1. Introduction

 Advanced angioplasty and stenting technology are two main therapeutic methods for treating cardiovascular disease. Although therapeutic percutaneous interventions have shown some good therapeutic efficacy of diverse vascular beds, such as the abdominal aorta and the iliac arteries [1, 2], the superficial femoral artery (SFA) restenosis and occlusion is of frequent occurrence after these interventions [3]. Additionally, it has been reported that vascular restenosis, as a critical complication after these procedures, is secondary to intimal hyperplasia (IH) [4]. Proliferation of VSMCs is a hallmark of the early pathologic appearance of IH [5, 6]. Given this, inhibition of VSMCs proliferation is the key to prevention and treatment of IH.

The p21-activated kinases (PAKs) are a family of serine/threonine kinases that are major effector proteins for the Rho GTPases Cdc42 and Rac, which are important for cell morphology and cytoskeletal reorganization [7, 8], as well as various cell processes including proliferation, migration, and survival [9–11]. Among them, p21-activated kinase 4 (PAK4) is the most unique and profoundly studied member. PAK4 is expressed at low levels in the majority of normal adult tissues and accumulating documents have reported that the aberrant expression of PAK4 is closely related to the diverse cancers, such as glioma, breast cancer, colon and gastric cancers, and prostate cancer [12–14]. Moreover, high expression of PAK4 is closely associated with cell proliferation, migration, invasion of ovarian cancer cell, and poor prognosis in patients [15]. Of significance, it has been reported that PAK4 plays an important role in the cell cycle through regulating the level of p21, a key member of the cyclin-dependent kinase- (CDK-) inhibitory protein family, in fibroblasts [16]. Additionally, PAK4 is highly expressed in embryonic stage, and knock-out of PAK4 would result in embryonic lethality, accompanied by anomalies in the heart and placenta and defects in vascular system [17, 18]. However, till date, there is no documented evidence of its pathological significance in VSMCs proliferation.

In the present study, we investigate whether PAK4 is involved in vascular restenosis using vascular samples from patients that underwent angioplasty of SFA and cell proliferation of VSMCs.

## 2. Materials and Methods

### 2.1. Ethics Statement

All procedures involving the use of human tissue were approved by the Ethics Committee of Changhai Hospital Second Military Medicine University, according to all ethical principles including the World Medical Association Declaration of Helsinki and the local legislation. All of the experiments were undertaken with the understanding and written consent of each subject according to the abovementioned principles.

### 2.2. Clinical Data and Tissue Specimens

The SFA samples were collected from 2014 to 2015 at Changhai Hospital Second Military Medicine University (experimental group, patients underwent percutaneous transluminal angioplasty (PTA) treatment of SFA, *n* = 3; control group, donors without clinical SFA restenosis, *n* = 3). Patients were treated in the standard manner of our practice. The three patients showed clinical restenosis and lower-limb necrosis was after postprocedure 10, 13, and 15 months, respectively. The SFA restenosis samples were harvested through amputation above the knee. The control SFA samples were obtained from the corresponding region of donors without clinical SFA restenosis. The SFA samples were promptly washed with PBS and resected longitudinally by the surgeon. Parts of the samples were stored immediately in −80°C for qRT-PCR assay and western blot analysis. The remaining samples were embedded with paraffin and prepared for further H&E staining. The inclusion criteria for the experimental participants were as follows: (1) CT angiography (CTA) showing that SFA restenosis occurred after the PTA treatment; (2) being willing to participate in the study. Exclusion criteria included CT angiography (CTA) showing SFA restenosis after the other surgery besides PFA treatment.

### 2.3. Cell Culture

Human vascular smooth muscle cell line T/G HA VSMC (ATCC® number: CRL-1999™) was purchased from American Type Culture Collection. Cells were cultured in Ham's F12K medium with 2 mM L-glutamine supplemented with 10% fetal bovine serum (FBS) at 37°C in a humidified incubator with 5% CO_2_. Then, cells were trypsinized, transferred to 10 cm tissue culture dishes, and cultured to subconfluence. Cells at passages 4–8 were used. Cells overexpressing PAK4 were obtained by transfection with PAK4 ORF expression clone vector, using Lipofectamine 2000 (Invitrogen) according to the manufacturer's protocol. For further experiments, cells were cultured in 6-well or 96-well plates with serum-free medium for 24 h.

### 2.4. Quantitative Real-Time PCR

Total RNA was isolated using TRIzol reagent (Invitrogen) according to the manufacturer's protocol. For the reverse transcription reaction, TaqMan MicroRNA Reverse Transcription Kit (Applied Biosystems) was applied. qRT-PCR was carried out in 7500 Fast Real-Time PCR System (Applied Biosystem) by using SYBR Green Universal Master Mix (Roche). Briefly, the following PCR program was performed: 50°C for 2 min and 95°C for 5 min, followed by 40 cycles for 15 s at 95°C and 56°C for 45 s. The sequence information is as follows: PAK4, forward primer: 5′-TCCCCCTGAGCCATTGTG-3′ and reverse primer: 5′-TG ACCTGTCTCCCCATCCA-3′; *β*-actin, forward primer: 5′-CTA TCGGCAA TGAGCGGTTC-3′ and reverse primer: 5′-GATCTTGATCTTCATGGTGCTAGG-3′. Mature PAK4 mRNA levels in cells were measured by 2^−ΔΔCT^ method, with *β*-actin as an internal control.

### 2.5. Cell Proliferation Assay

Cell proliferation of VSMCs was detected via cell count technique. In brief, cells were seeded in 96-well plates with a density of 1 × 10^**4**^ cells/mL and incubated for 24, 48, and 72 h. Then, cell proliferation was assessed by direct cell count with a Coulter Counter. The experiments were performed three times.

### 2.6. BrdU Incorporation Assay

The viability of the VSMCs was determined by 5-Bromo-2-deoxyUridine (BrdU) assay according to the manufacturer's instructions. Cells transfected with PAK4 or control vectors were seeded in sterile 96-well culture plates at a density of 2 × 10^5^ cells per well with serum-free medium and incubated for 48 or 72 h. Then, cells were incubated in medium containing a final concentration of 10 *μ*M BrdU for 2 h. Subsequently cells were washed and fixed with 2% paraformaldehyde solution for 25 min at room temperature and then washed three times with PBS to discard the culture medium. After observation, pictures were taken randomly in diverse views under a fluorescent inverted microscope.

### 2.7. Cell Cycle Assay

Flow cytometry was conducted to analyze cell cycle. The cells were collected, trypsinized, and fixed with 75% methanol at −20°C overnight and then washed in PBS three times and incubated with PBS containing 10 ng/mL propidium iodide (PI), 100 *μ*g/mL RNase A, and 0.2% Triton X-100 for 30 min at 4°C in the dark. DNA content was monitored using a cell sorter (FACSCalibur, BD).

### 2.8. Western Blot

The PAK4 protein levels of VSMCs and clinical tissues were assayed by western blot technology. The cells were grown in 6-well culture dishes to 70% confluence. Cells and tissues were lysed with RIPA buffer. After 15 min incubation, lysates were centrifuged at 12,000 g for 15 min, and the collected protein was loaded onto 10% sodium dodecyl sulfate-polyacrylamide gel (SDS-PAGE) and transferred onto polyvinyl difluoride (PVDF) membrane. The membranes were blocked with skimmed milk powder in Tris-buffered saline and then incubated with primary anti-PAK4 (1 : 500), anti-p21 (1 : 400), anti-p-Akt (1 : 1000), anti-Akt (1 : 300), and anti-*β*-actin (1 : 200) at 4°C overnight, followed by incubation with horseradish peroxidase-conjugated anti-rabbit secondary antibody (1 : 5000). The bands were visualized with the enhanced chemiluminescence plus system (ThermoFisher Scientific).

### 2.9. Histochemistry and Immunohistochemistry

Vascular tissues were washed with 0.9% saline, fixed with 4% neutral buffered paraformaldehyde and embedded in 10% paraffin. The intimal and medial lesion size were assessed by H&E staining and photographed. Stained specimens were assessed by a pathologist with a light microscope (Leica DM 6000 B; Leica Microsystems, Germany).

### 2.10. Statistical Analysis

All experiments were repeated at least three times in this study. Data were presented as mean ± standard deviation (SD). Statistically significant differences between two groups were performed via *t*-test. A *P* value < 0.05 was considered as statistical significance.

## 3. Results

### 3.1. PAK4 Expression in Human Vascular Walls with IH

**Figure 1:**
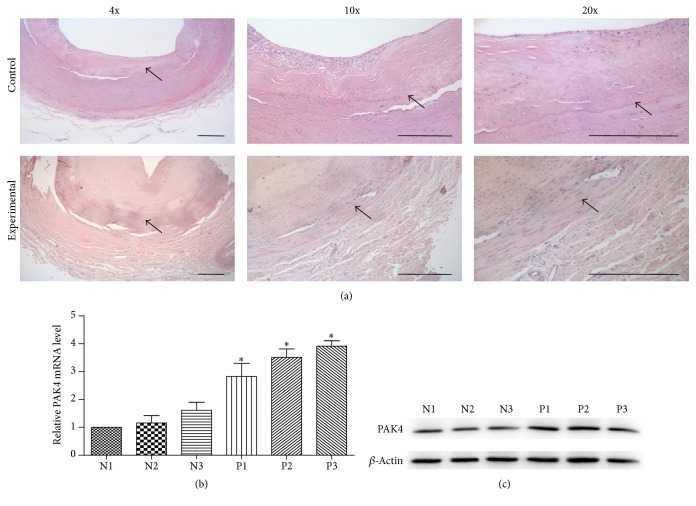
*PAK4 involved in IH in human superficial femoral artery (SFA).* (a) Representation of vascular walls in normal people and patients with H&E staining (magnification ×4, ×10, and ×20, resp.). Normal, human SFA samples collected from normal people. Surgery, human SFA samples collected from patients that underwent percutaneous transluminal angioplasty (PTA) treatment of SFA. The arrows indicated the vascular intima. (b) qRT-PCR analysis of PAK4 mRNA levels in control group (N1–N3) and experimental group (P1–P3). (c) Western blot analysis of PAK4 protein levels in control group (N1–N3) and experimental group (P1–P3). (*n* = 3, data are means ± SD, ^*∗*^*P* < 0.05 versus N1).

The degree of IH was evaluated morphologically using H&E staining. As shown in Figure 1(a), the intima layer was dramatically thickened in experimental group compared with the control group. To define the clinical functional role of PAK4, we detected the mRNA and protein expression levels of PAK4 in tissues with IH that arose from angioplasty. qRT-PCR indicated that, compared with the control samples, IH significantly increased the mRNA level of PAK4 (Figure 1(b)). Consistent with these, the western blot results showed that the expression of PAK4 protein was remarkably unregulated by IH (Figure 1(c)). Further details of the patients were listed in Table 1. As shown, there was no significant difference in complications of cardiac and cerebrovascular diseases between the two independent cohorts. These results demonstrated that PAK4 may play a vital role in the pathogenesis of IH originated from angioplasty.

### 3.2. PAK4 Facilitates Vascular Smooth Muscle Cells' Proliferation

**Figure 2:**
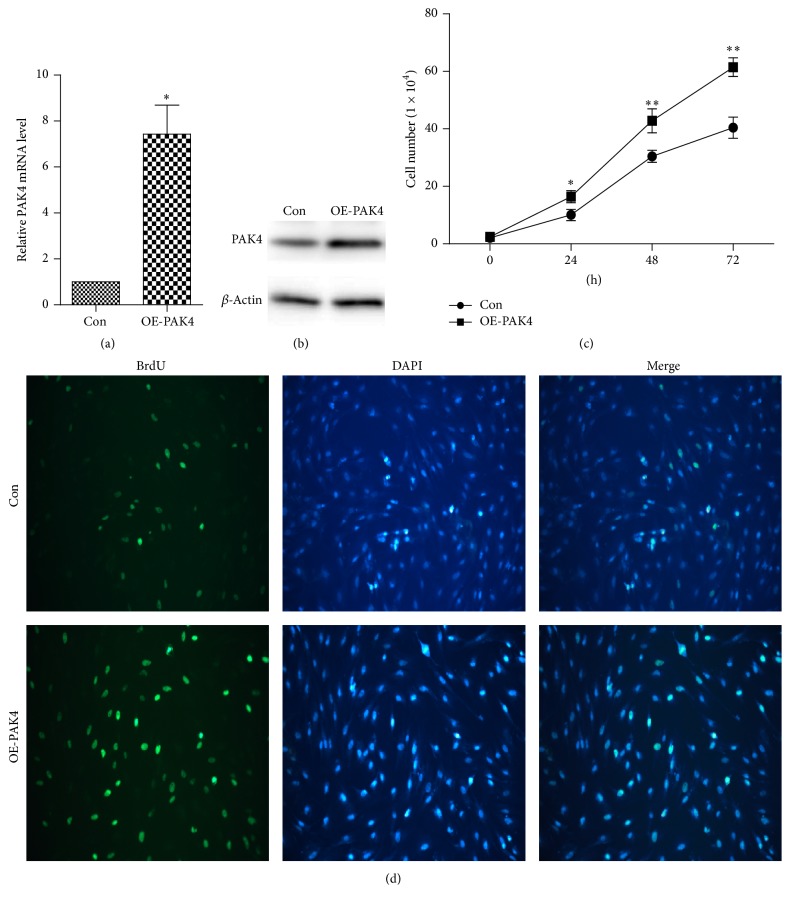
*PAK4 promotes vascular smooth muscle cells' (VSMCs) proliferation.* (a) qRT-PCR analysis of PAK4 mRNA levels in OE-PAK4 VSMCs and the control group. (b) Western blot analysis of PAK4 protein levels in OE-PAK4 VSMCs and the control group. (c) Cell growth detected by cell count technique. (d) Cell proliferation was detected by BrdU incorporation assay (magnification ×200). BrdU (green fluorescence) and DAPI (blue fluorescence). The stained images were digitally merged. (OE-PAK4: overexpression of PAK4 VSMCs group. Con: control VSMCs group; *n* = 3, data are means ± SD, ^*∗*^*P* < 0.05, ^*∗∗*^*P* < 0.001 versus Con).

To gain insight into the pathobiological involvement of PAK4 in IH, the VSMCs of PAK4 overexpression were constructed. We identified the efficiency of transfection with qRT-PCR and western blot. As a result, PAK4 mRNA and protein levels were significantly higher in VSMCs of PAK4 overexpression than the control cell line transfected with mock-vehicle (Figures 2(a) and 2(b)). Further, cell count technique and BrdU incorporation assay were performed to detect cell proliferation. As shown in Figure 2(c), PAK4 overexpression in VSMCs resulted in faster growth than that in control group, especially when cells were incubated for 48 h (42.80% versus 30.46%, *P* < 0.001). Similarly, in BrdU assay, PAK4 overexpression in VSMCs obviously accelerated cell proliferation at 48 h (Figure 2(d)). These data indicated that PAK4 could facilitate the proliferation of VSMCs.

### 3.3. PAK4 Promotes Vascular Smooth Muscle Cells' Cycle Progression

**Figure 3:**
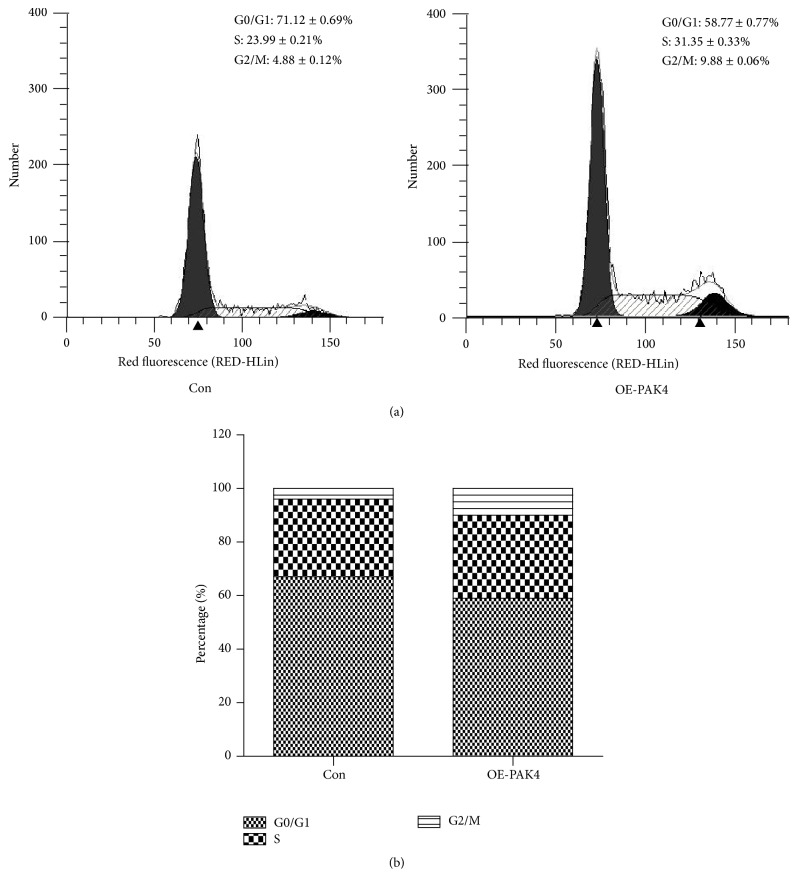
*PAK4 regulates vascular smooth muscle cells cycle progression.* (a) PAK4 expression promotes VSMCs in the S phase of the cell cycle analyzed by flow cytometry (peak image). (b) PAK4 expression promotes VSMCs in the S phase of the cell cycle analyzed by flow cytometry (bar image). OE-PAK4: overexpression of PAK4 VSMCs group. Con: control VSMCs group.

The effects of PAK4 on cell cycle progression were also analyzed. Flow cytometry analysis revealed that overexpression of PAK4 obviously increased the number of VSMCs in the G2/M phase and S phase (4.88 ± 0.12% versus 9.88 ± 0.06%, *P* < 0.001; 23.99 ± 0.21% versus 31.35 ± 0.33%, *P* < 0.001) (Figures 3(a) and 3(b)). The percentage of cells in G0/G1 phase decreased from 71.12% to 58.77% after PAK4 overexpressed in VSMCs (Figures 3(a) and 3(b)). This data suggested that PAK4 might promote the progression of cell cycle from G0/G1 phase into S phase, which further contributed to the proliferation of VSMCs.

### 3.4. PAK4 Modulates p21 Expression and Akt Activation

**Figure 4:**
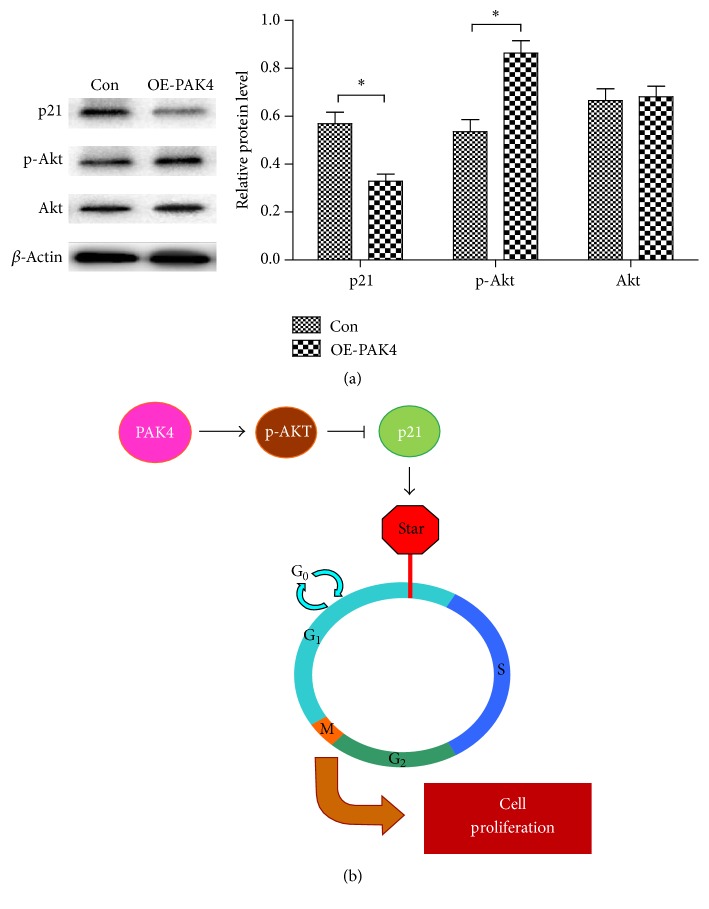
*The mechanism of PAK4 modulating VSMCs proliferation.* (a) Western blot analysis of p21, p-Akt, and Akt protein levels in OE-PAK4 VSMCs and control group. The protein expression levels were normalized to *β*-actin. ^*∗*^*P* < 0.05. (b) A model of PAK4 mediating VSMCs proliferation. PAK4 expression induces the activation of Akt signaling, which further attenuates the level of p21. The decreased p21 level promotes G0/S transition in cell cycle progression and then contributes to VSMCs proliferation (*n* = 3).

To define the potential mechanism by which PAK4 signaling confers regulating cell cycle progression, we investigated the expression level of several putative cell cycle-related factors in VSMCs. In the overexpressing PAK4 group and control group, we detected the protein expression levels of p21, p-Akt, and Akt by using western blot analysis. As the results showed, the level of p21 protein was dramatically low in PAK4 overexpressed VSMCs group compared to the control cell line (Figure 4(a)). At the same time, overexpression of PAK4 notably increased the phosphorylation of Akt, while it had no effect on total protein levels of Akt. The data together indicated that PAK4 might mediate cell cycle progression through regulating the expression of p21 and the activation of Akt, further contributing to VSMC proliferation (Figure 4(b)).

## 4. Discussion

Percutaneous transluminal angioplasty (PTA) and stent implantation have become a top option of therapeutic schedule in atherosclerotic diseases, which is mainly manifested by peripheral arterial occlusive disease. However, PTA contributes to the occurrence of restenosis, especially in the SFA, which would dramatically decrease the efficacy of treatment [19]. Thus, it is of great importance to inhibit post-PTA restenosis. It has been widely accepted that the VSMCs proliferation was the pathological basis of IH, further resulting in vascular stenosis [20, 21].

The PAK proteins family was involved in diverse cellular activities, which was classified into group I (PAK1-3) and group II (PAK4-6), based on the domain organization and regulatory properties [22]. PAK4, the prototype of group II PAKs, has been indicated to be involved in a variety of cellular processes, including cell proliferation, cell mobility, and cell cycle regulation [16, 23]. Cell cyclin-dependent kinase inhibitor p21 plays an important role in suppressing cell cycle progression. Previous report indicated that knockdown of PAK4 obviously prohibited breast cancer cell proliferation [22]. However, the role of PAK4 in VSMCs proliferation has not been explored. Here, clinical studies reveal that IH results in an enhancement of PAK4 expression. Therefore, it is not surprising that PAK4 has effect on cell proliferation in VSMCs. In our study, VSMCs proliferation was increased with the overexpression of PAK4, which confirmed the speculation.

Improper regulation of cell cycle could be the vital factor of aberrant cell proliferation. p21, a member of CDK-inhibitory protein family, suppresses cell cycle process through the Ras-Raf-MEK-ERK signaling pathway [24, 25]. p21 is a short-lived protein, of which the level is modulated by ubiquitin-independent proteasome degradation process [26]. Diverse signaling pathways could contribute to the alternation of p21 level via different mechanism. Importantly, it has been documented that PAK4 plays a significant role in the initiation of cell cycle via regulating the cell cycle regulatory protein p21 [16]. In the present study, we demonstrated that overexpression of PAK4 arrested the cell cycle in S phase by flow cytometer. And further western blot results revealed that the expression of p21 protein was dramatically downregulated with the overexpression of PAK4 in VSMCs. Our study reported the integrated roles for PAK4 and p21 in VSMCs, which are of great importance in VSMCs proliferation and the development of restenosis.

It is well established that Akt plays an important role in regulating cell cycle progression and cell survival by targeting at diverse downstream targets [27]. Moreover, in VSMCs, the Akt signal was proved to be closely related to controlling VSMCs proliferation, at least partly, through modulating p21 level [28]. Besides, a relationship between Akt and PAK4 has also been previously demonstrated. PAK4 has been shown to be controlled by miRNA-433 and subsequently attenuates Akt signaling, resulting in regulating the proliferation of hepatocellular carcinoma (HCC) cells. Following these data, we speculated that PAK4 might have some effect on p21 expression via regulating the activity of Akt signaling in VSMCs. This notion was validated by our findings that overexpression of PAK4 in VSMCs remarkably decreased the level of p21 and enhanced the activation of Akt simultaneously and further mediate cell cycle progression and cell proliferation. Thus, our findings have provided experimental evidence to support that PAK4 exerts the effects cell proliferation and cell cycle progression on VSMCs via regulating Akt signaling and the downstream factor p21.

## 5. Conclusions

In conclusion, we provided the direct evidence that PAK4 was involved in IH triggered by angioplasty with clinical data. The results demonstrated that PAK4 mediated IH via promoting cell proliferation of VSMCs. Further, we sought to verity the underlying mechanism of this effect, and the data suggested that PAK4 increased VSMCs proliferation through activating the Akt signaling and downregulating the expression of p21 protein, further resulting in G0/S phase transition of cell cycle progression. These findings contribute to the clarification of the crucial role of PAK4 in IH and might provide potential therapeutic targets for restenosis.

## Figures and Tables

**Table 1:** Patient characteristics of the 6 samples.

Demographics	Control group			Experimental group		
	1	2	3	4	5	6
Gender	Male	Female	Male	Male	Male	Female
Age (year)	76	79	74	78	75	77
Hypertension	+	+	+	+	+	+
Coronary disease	−	+	−	+	−	−
Diabetes	−	−	−	−	−	−
Atrial fibrillation	−	−	−	−	−	−
Chronic renal insufficiency	−	−	−	−	−	−
Hyperlipemia	−	+	−	+	−	−
Cerebral infarction	−	−	−	−	+	−
Smoking history	+	−	+	+	+	−
